# Local Responses and Systemic Induced Resistance Mediated by Ectomycorrhizal Fungi

**DOI:** 10.3389/fpls.2020.590063

**Published:** 2020-12-14

**Authors:** Steven Dreischhoff, Ishani S. Das, Mareike Jakobi, Karl Kasper, Andrea Polle

**Affiliations:** Forest Botany and Tree Physiology, University of Göttingen, Göttingen, Germany

**Keywords:** ectomycorrhiza, systemic resistance, mycorrhiza, plant defense, phytohormone, chitin, herbivores

## Abstract

Ectomycorrhizal fungi (EMF) grow as saprotrophs in soil and interact with plants, forming mutualistic associations with roots of many economically and ecologically important forest tree genera. EMF ensheath the root tips and produce an extensive extramatrical mycelium for nutrient uptake from the soil. In contrast to other mycorrhizal fungal symbioses, EMF do not invade plant cells but form an interface for nutrient exchange adjacent to the cortex cells. The interaction of roots and EMF affects host stress resistance but uncovering the underlying molecular mechanisms is an emerging topic. Here, we focused on local and systemic effects of EMF modulating defenses against insects or pathogens in aboveground tissues in comparison with arbuscular mycorrhizal induced systemic resistance. Molecular studies indicate a role of chitin in defense activation by EMF in local tissues and an immune response that is induced by yet unknown signals in aboveground tissues. Volatile organic compounds may be involved in long-distance communication between below- and aboveground tissues, in addition to metabolite signals in the xylem or phloem. In leaves of EMF-colonized plants, jasmonate signaling is involved in transcriptional re-wiring, leading to metabolic shifts in the secondary and nitrogen-based defense metabolism but cross talk with salicylate-related signaling is likely. Ectomycorrhizal-induced plant immunity shares commonalities with systemic acquired resistance and induced systemic resistance. We highlight novel developments and provide a guide to future research directions in EMF-induced resistance.

## Introduction

Plants live in close relationship with microbes, which colonize their hosts as symbiotrophic, saprotrophic or pathogenic organisms (Bonfante and Anca, 2009; Vandenkoornhuyse et al., 2015). An important example is the beneficial interaction between certain soil fungi and plant roots, leading to the formation of a new organ, the mycorrhiza (from Greek μýκη*ς* mıkçs, “fungus,” and ßζα rhiza, “root”). The mycorrhizal symbiosis is well characterized by a bidirectional exchange of nutrients (Smith and Read, 2008). The fungus receives photosynthesis-derived carbohydrates from the plant and supplies essential, often rarely available nutrients like nitrogen or phosphorus from the soil to the plant (van der Heijden et al., 2015; Nehls and Plassard, 2018).

Mycorrhizal symbiosis enhances the performance of plants (Smith and Read, 2008) and, thus, most likely drastically facilitated the evolution of land plants (Wang et al., 2010). Approximately 85 % (˜340,000 species) of all plant species are colonized by mycorrhizal fungi (˜50,000 species) (van der Heijden et al., 2015; Brundrett and Tedersoo, 2018; Genre et al., 2020). The most ancient and widely spread symbiosis is formed by arbuscular mycorrhizal fungi (AMF) (Bonfante and Anca, 2009; Martin et al., 2018). In forests of the temperate and boreal zone, ectomycorrhizal symbioses with the roots of tree species are predominant (Brundrett, 2009). Ectomycorrhizal fungi (EMF) have evolved independently multiple times from saprotrophic clades, making EMF no homogenous group (Martin et al., 2016; Genre et al., 2020). EMF and AMF are the most well studied groups among mycorrhiza-forming fungi, however, exhibiting different lifestyles. While AMF form hyphopodia to invade the plant and grow inside cortical root cells, EMF cover the root tip with a hyphal mantle and grow between the root epidermis and outer layers of cortical cells, forming the Hartig net (Bonfante and Anca, 2009). Both AMF and EMF generate extraradical hyphae as the main structures for nutrient uptake from soil.

There is now growing awareness that mycorrhizas do not only improve plant nutrition but also enhance plant resistance against abiotic and biotic cues. Resistance is the ability of a plant to restrict the growth and development or the damage caused by a specific pest or pathogen. Resistance can be achieved by activation of defense mechanisms or is the result of tolerance, i.e., the ability to endure the stress (Larcher, 1995). The term “mycorrhiza-induced resistance” (MIR) has been used to describe this phenomenon for the interaction of a mycorrhizal fungus with a host plant (Cameron et al., 2013; Mauch-Mani et al., 2017). MIR shares similarities with both systemic acquired resistance (SAR), induced after pathogen attack, while induced systemic resistance (ISR) is conferred by beneficial soil microbes. In this review, we focus on ectomycorrhiza-induced systemic resistance, which is a rapidly expanding research area. We define systemic effects as those effects that occur in distal tissues (here leaves) that are not in direct contact with the mycorrhizal fungus, while local responses occur in tissues (here roots) in contact with the EMF. We discuss local responses to EMF colonization, leading to long-distance signaling, systemic transcriptional rewiring and metabolic changes induced by EMF. We address the role of phytohormones in MIR and discuss commonalities with SAR and ISR. Since MIR by EMF is an emerging field, we also include examples for MIR induced by AMF highlighting similarities in defense activation.

## A Glimpse on Systemic Resistance in Plants—SAR and ISR

The two major types of systemic resistance intensely studied in plant microbial interactions are SAR (Spoel and Dong, 2012) and ISR (Pieterse et al., 2014). SAR and ISR are based on distinct phytohormonal signals. SAR describes defenses against (hemi-)biotrophic pathogens activated after local challenge by a pathogen in systemic, uninfected tissues. The SAR signaling cascade is triggered by microbe-associated molecular patterns (MAMPs) leading to MAMP-triggered immunity or triggered by pathogen effectors leading to effector-triggered immunity (Jones and Dangl, 2006). Subsequently, the defense in systemic uninfected tissues is induced in an SA dependent manner and acts against a broad range of pathogens (Vlot et al., 2009; Spoel and Dong, 2012). Various compounds have been proposed as potential signals for SAR activation. For instance, methyl salicylate is a phloem-mobile compound that can be transported to systemic plant parts, where it is hydrolyzed to the bio-active SA to induce resistance (Park et al., 2007). For defense induction and in addition for attracting predators of herbivores, methyl SA might also act as a volatile signal (Shulaev et al., 1997; Koo et al., 2007; Ament et al., 2010; Rowen et al., 2017). Recently, the non-proteinogenic amino acid pipecolic acid (Pip) and its derivative N-hydroxypipecolic acid have been identified as essential for SAR signaling (Návarová et al., 2012; Chen et al., 2018; Hartmann et al., 2018; Wang et al., 2018). The mobile signals activate MAPK (MITOGEN-ACTIVATED PROTEIN KINASE) cascades (Conrath et al., 2015) and induce the expression of pathogenesis-related (PR) proteins, especially PR1 (PATHOGENESIS-RELATED 1) involving antagonistic key regulators NPR1 and NPR3/4 [NON-EXPRESSER OF PR GENES (Ding et al., 2018)]. Other compounds invoked as mobile SAR signals are azaleic acid (a C_9_ lipid peroxidation product), lipid transfer proteins, and the diterpene dihydroabietinal (Vlot et al., 2017). Ultimately, an enhanced defense is achieved either through direct defenses (e.g., callose deposition) or trough priming, whereby the plant exhibits stronger defenses toward a secondary infection (Conrath et al., 2006; Jung et al., 2009, 2012; Pieterse et al., 2014; Mauch-Mani et al., 2017).

In contrast to SAR induced by pathogens, ISR is conferred by beneficial microbes. They interact with roots and make the whole plant more resistant or tolerant against stressors. The picture for ISR is less specific than for SAR because different microbial species might recruit different compounds for ISR signaling (Haney et al., 2018). In general, jasmonic acid (JA) and its derivatives, in particular JA-Isoleucine (JA-Ile) are the key phytohormones and their signaling pathways are modulated by either ethylene (defense against necrotrophic pathogens) or abscisic acid (against herbivores) (Pieterse et al., 2012). JAZ (JASMONATE-ZIM-DOMAIN PROTEIN), which stabilize the JA receptor COI1 (CORONATINE INSENSITIVE 1), and MYB (MYB DOMAIN PROTEIN) transcription factors are essential in ISR. Similar to SAR, more than one component might act as a long-distance signal (see section “Long-Distance Signaling in Systemic Resistance—Tapping Around in the Dark”). At the cellular level, the pathways for systemic defenses, ISR and SAR often appear to be regulated antagonistically. When SA signaling is upregulated, JA signaling is suppressed, implying trade-off for the resistance against necrotrophic pathogens when the defense against biotrophic pathogens is upregulated and vice versa (Pieterse et al., 2012).

## Shedding Light on Ectomycorrhizal Induced Defenses

### Defense Signaling in Local Root Tissue Interacting With EMF Unveils Commonalty With Pathogen-Triggered Responses

In the process of establishing an active symbiosis, host plant and EMF exchange an array of molecules with different properties, e.g., flavonoids, auxin, and secreted proteins, etc. (Felten et al., 2009; Garcia et al., 2015). Genome, transcriptome, and secretome analyses of EMF from distant phyla (basidiomycota: *Laccaria bicolor*, ascomycota: *Tuber melanosporu* and *Cenococcum geophilum* (Vincent et al., 2012; Doré et al., 2015; Kohler et al., 2015; Pellegrin et al., 2015; de Freitas Pereira et al., 2018) uncovered a huge battery of small secreted proteins, among which a subset was strongly up-regulated during mycorrhizal colonization of the host. Three mycorrhizal-induced small proteins, MiSSP7, 7.6, and 8 (named after their atomic mass in kDa) of *L. bicolor* were closer investigated and found to be essential for symbiosis establishment (Plett et al., 2011; Pellegrin et al., 2019; Kang et al., 2020).

In *Populus* × *canescens, Lb*MiSSP7 interacts locally with JAZ6 to stabilize this protein (Plett et al., 2014). JAZ6 is a key repressor of the F-box protein COI1, which is the receptor for JA-Ile, the active form of JA, in the SCF(COI1) complex (Thines et al., 2007). When COI1 binds JA-Ile, JAZ6 is degraded via the proteasome and the transcription of JA responsive genes is activated (Howe et al., 2018). Thus, by stabilizing *Populus* JAZ6 the JA signaling pathways is locally suppressed. Application of JA acts negatively on the establishment of symbiotic structures (Plett et al., 2014). Because of the JA-SA antagonism (see section “A Glimpse on Systemic Resistance in Plants—SAR and ISR”), this regulation is surprising as it may be intuitively expected to facilitate defenses against biotrophic fungi (including EMF). Plett et al. (2011) demonstrated that MiSSP7 also induces the transcription of auxin-responsive genes in root tissues.

Circumstantial evidence suggests that *Lb*MiSSP7.6 may also interfere with local plant immunity. *Lb*MiSSP7.6 interacts with two *Populus* Trihelix transcription factors (*Pt*Trihelix1 and *Pt*Trihelix2) in the nucleus of plant cells. The closest *Arabidopsis thaliana* homolog of *Pt*Trihelix2 is *At*ASR3 (ARABIDOPSIS SH4-RELATED3) (Kang et al., 2020), which is a phosphorylation substrate of MAPK4 and thus, may negatively regulate immunity. Furthermore, pattern-triggered immunity is negatively regulated through phosphorylation of *At*ASR3 by MAPK4 (Li et al., 2015).

Additional support for the modulation of immune responses by small secreted proteins comes from studies on the AMF-host interactions (*Glomus intraradices* with *Medicago truncatula*) (Kloppholz et al., 2011). The AMF fungal protein *Gi*SP7 (secreted protein 7) interacts with *Mt*ERF19 (ETHYLENE-RESPONSIVE FACTOR) transcription factor in the plant nucleus and interferes with *Mt*ERF19-related and, thus, ethylene-modulated defense (Kloppholz et al., 2011). Moreover, *Mt*ERF19 is induced by pathogens and is involved in activating defense against biotrophic pathogens (Kloppholz et al., 2011). Taken together, these examples show that mycorrhizal fungi interact with parts of the plants defense local machinery involving small secreted protein.

Not only small secreted protein could be responsible for the initiation of defense induction, but also a number of other metabolites. It is known for AMF that chitin oligomers and lipochitooligosaccharides are part of Myc factors, which are used for communication with their host (Maillet et al., 2011; Sun et al., 2015). These compounds are also produced by the EMF *L. bicolor* (Cope et al., 2019). In poplar, lipochitooligosaccharides from *L. bicolor* activate the common symbiosis pathway including calcium-spiking (Cope et al., 2019), which plays a role in activating defense responses to microbes (Yuan et al., 2017). Lipochitooligosaccharides were also found to modulate host immunity (Limpens et al., 2015). Furthermore, chitin and chitin-related components (e.g., chitosan) are known elicitors (MAMPs) for plant defense (Boller and Felix, 2009). Chitosan formulations have been applied as a biological control agents to leaves and roots to boost plant defenses (El Hadrami et al., 2010; Pusztahelyi, 2018) but their mode of action is unclear.

**FIGURE 1:**
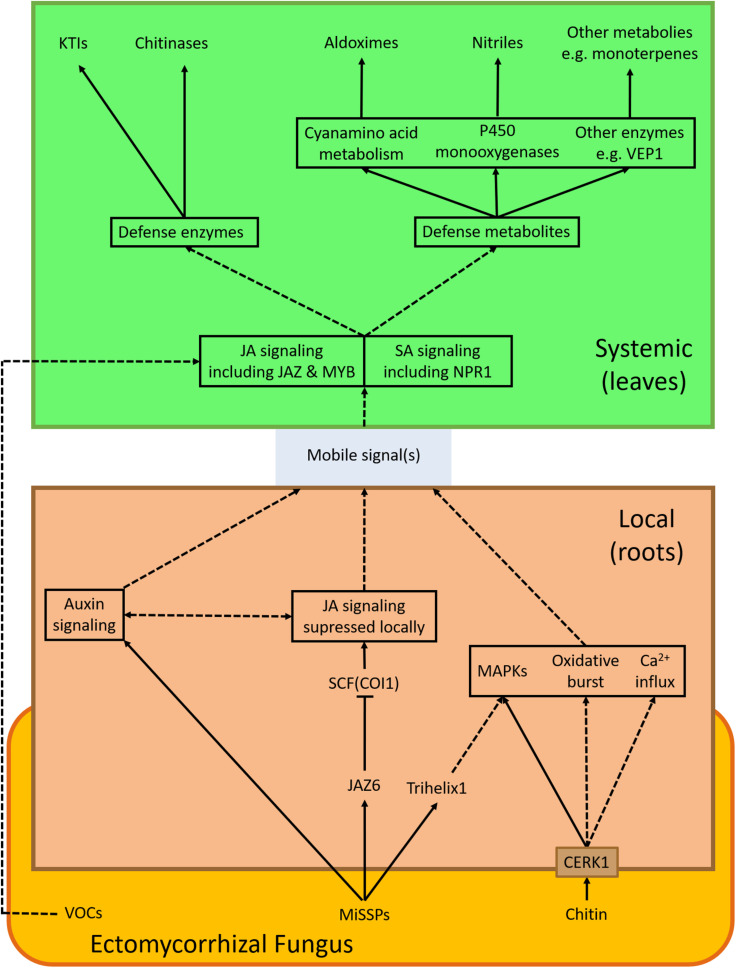
EMF-induced signaling cascade(s). The scheme summarizes signaling components discussed in this review displaying processes induced by an ectomycorrhizal fungus (red) in the root (brown) and in leaves (green). Arrows show interactions/connections (full lines: direct evidence, dashed lines: speculative). VOCs—Volatile organic compounds; JAZ6—JASMONATE-ZIM-DOMAIN PROTEIN 6; CERK1—CHITIN ELICITOR RECEPTOR KINASE 1; SCF(COI1)— CORONATINE INSENSITIVE 1—Skp, Cullin, F-box containing complex; MAPKs—mitogen-activated protein kinases; JA—jasmonic acid; MYB—MYB DOMAIN PROTEIN; KTIs—Kunitz trypsin inhibitors; VEP1—VEIN PATTERNING 1.

Chitin is a cell wall component of fungi but not of plants. Novel results assign a crucial role to chitin in fungal perception and defense stimulation (Zhang et al., 2015; Vishwanathan et al., 2020). When Arabidopsis roots were treated with chitin enhanced protection against leaf herbivory was observed similar to that found in response to *L. bicolor* inoculation of Arabidopsis roots (Vishwanathan et al., 2020). This finding shows that—at least part of—MIR by EMF does not require formation of a functional mycorrhiza because Arabidopsis is a non-host to mycorrhizal fungi. This result further shows that chitin, an abundant compound in many potentially hazardous organisms (fungi, insects), is sufficient for the defense induction. The plant chitin receptor CHITIN ELICITOR RECEPTOR KINASE 1 (CERK1) (Miya et al., 2007) is well known for its central role in mediating plant immunity (Gong et al., 2020). In Arabidopsis knock-out mutants *cerk1-2* MIR induced by EMF was abolished (Vishwanathan et al., 2020; Figure 1), demonstrating the critical role of chitin in the non-host interaction of *Arabidopsis* with *L. bicolor*. Other components such as LYK4 and LYK5 forming a complex with CEKR1 necessary for defense induction (Xue et al., 2019), may also be involved but this assumption has yet to be experimentally tested. Upon chitin or *L. bicolor* exposure, the MAP kinase signaling cascade (MAPK 3, 4, and 6) was activated in Arabidopsis (Vishwanathan et al., 2020). MAPKs belong like calcium influx and oxidative burst to the microbial triggered immunity responses (Boller and Felix, 2009), suggesting that *L. bicolor* activates a general microbial defense pathway via chitin perception. In rice, CERK1 has also a function in defense signaling and AMF symbiosis (Zhang et al., 2015).

Chitin is released from fungal cell walls by plant chitinases as part of defenses against fungal pathogens (Sharma et al., 2011). It was first reported for the interaction of *Amanita muscaria* with *Picea abies* that EMF also induce chitinases in host roots (Sauter and Hager, 1989). Similarly Albrecht et al. (1994a, b) showed that chitinases are induced upon contact of the EMF *Pisolithus tinctorius* with *Eucalyptus globulus* and that the strength of this defense response correlated with the extent of colonization by the fungus. Many previous studies showed transient transcriptional activation of chitinases in concert with other defenses (e.g., metallothionein-like proteins and glutathione-*S*-transferases) when EMF interacted with host roots (Franken and Gnädinger, 1994; Johansson et al., 2004; Duplessis et al., 2005; Frettinger et al., 2007; Heller et al., 2008).

A number of studies indicate that host colonization by EMF activates local host defenses only transiently. For example, the transcription of defense genes was locally upregulated in birch roots during the formation of the ectomycorrhizal mantle and the Hartig net by *Paxillus involutus* (Le Quéré et al., 2005). In later developmental stages when the mycorrhiza was mature, plant defense genes were repressed (Le Quéré et al., 2005). In oak colonized by the EMF *Piloderma croceum*, genes of the phenylpropanoid metabolism were down-regulated (Tarkka et al., 2013). A recent study shows that the transcriptional responses in oak vary substantially depending on the ectomycorrhizal fungal species that is colonizing the root, but a common response induced by the tested EMF species was the reduction of defense gene transcript levels, when the roots had been colonized (Bouffaud et al., 2020). It is therefore possible that initially fungal MAMPs induce defenses, which are subsequently suppressed by mechanisms similar to those employed pathogenic fungi (Barsoum et al., 2019).

Altogether, these studies highlight that EMF locally trigger (a subset of) plant defenses against fungal pathogens, at least during the initial stages of colonization. Chitin signaling is required to elicit systemic responses in distant tissues. Intriguing questions for future research are whether MIR is part of the universal non-host response of plants to microbes or whether MIR in a functional mycorrhiza as the result of compatible EMF-host interactions has additional facets.

### Long-Distance Signaling in Systemic Resistance—Tapping Around in the Dark

Mobile inter-organ signaling is required to achieve MIR in systemic tissues. The most direct and fastest connection between mycorrhizal roots and the shoot is the xylem. In addition to its function in water and mineral nutrition transport, the composition of the xylem sap is characterized by a plethora of compounds such as phytohormones, proteins, peptides, and amino acids, etc. (Shabala et al., 2016). In response to nodulation by rhizobia or symbiosis with AMF, specific small peptides (CLE) have been found (Okamoto et al., 2013; Le Marquer et al., 2019), which are part of the plant autoregulation of symbiotic interactions (Wang et al., 2018). Given the similarities of the genetic make-up of root symbiotic interactions for EMF, AMF, and rhizobia (Cope et al., 2019), it is tempting to speculate that CLE peptides may also signal the root mycorrhizal status in EMF plants. However, to date neither peptides nor phytohormones or other molecules have been identified in xylem sap that were functionally linked with MIR in EMF plants.

MIR influences the performance of phytophagous insects (Pozo and Azcón-Aguilar, 2007). Therefore, it is conceivable that JA(-derivatives), which are known to mount defenses against wounding and insect feeding (Zhang and Hu, 2017), play a role in long-distance signaling of MIR. JA-derived molecules such methyl-JA can be transported in both the xylem and the phloem (Thorpe et al., 2007). Mutants of tomato, which are unable to mount systemic defenses, revealed that the systemic wound response requires local JA biosynthesis and the ability to perceive a JA signal systemically (Schilmiller and Howe, 2005).

Vascular transport of mobile signals has most intensely been studied for SAR. The phloem was identified as the major signaling route (Shah and Zeier, 2013). Upon interaction with biotrophic pathogens or virulence factors, compounds such as the methyl ester of salicylic acid (Dempsey and Klessig, 2012), JA (Truman et al., 2007), and pipecolic acid (Shah and Zeier, 2013) accumulate in the vasculature and were able to induce SAR independently of other compounds. Azelaic acid (Jung et al., 2009), a glycerol-3-phosphate-derived molecule (Chanda et al., 2011) and the abietane diterpenoid dehydroabietinal (Chaturvedi et al., 2012) are bound to the lipid transport protein DIR1 (DEFECTIVE IN RESISTANCE1) for transport through the vasculature, leading to SAR induction (Isaacs et al., 2016). Most of the potential SAR signaling molecules accumulate in petiole exudates (Maldonado et al., 2002; Thorpe et al., 2007; Truman et al., 2007; Jung et al., 2009; Chanda et al., 2011; Sato et al., 2011; Chaturvedi et al., 2012; Champigny et al., 2013; Isaacs et al., 2016). Feeding petiole exudates of SAR-induced wildtype Arabidopsis to transgenic lines, unable to express the signaling compound glycerol-3-phosphate or *DIR1*, recovered SAR in the mutants (Chanda et al., 2011; Isaacs et al., 2016). In poplar, SA or methyl-SA can induce resistance in systemic tissues (Li et al., 2018). These phytohormones are also required for the activation flavan-3-ols synthesis as defense against rust fungi (Ullah et al., 2019). Whether SA or its derivatives also play a role in the transmission of EMF-induced signals in trees is still unknown.

In addition to the classical pathways through xylem and phloem for the directed transport of molecules, volatile organic compounds (VOCs) are undirected aerial signals, serving inter-kingdom communication between plants and fungi (Werner et al., 2016; Schulz-Bohm et al., 2017). EMF emit a rich spectrum of VOCs, dominated by mono- and sequiterpenes (Müller et al., 2013). Among these VOCs, β-caryophyllene mounts plant defenses against bacterial pathogens (Huang et al., 2012; Hammerbacher et al., 2019). Furthermore, EMF influence the VOC emission pattern of mycorrhizal poplar, leading for example to slightly suppressed ocimene levels (Kaling et al., 2018). The perception of VOCs and stimulation of defenses can be amplified, activating SAR from plant to plant (Wenig et al., 2019). Since direct evidence for genes responsive to VOCs and EMF is missing, we screened the literature for genes regulated in response to VOCs (Godard et al., 2008; Riedlmeier et al., 2017; Lee et al., 2019) overlapping with those responsive to EMF (Luo et al., 2009; Kaling et al., 2018; Table 1). Notably, many of these genes are involved in JA signaling and play roles in wounding or pathogen defense (*JAZ1, JAZ7, JAZ8, WRKY40, β-1,3-ENDO-GLUCANASE, SIS, CYP94B1, and GSTU1*; Table 1). These observations suggest that long-distance signaling by VOCs should be taken into account in future studies of systemic defense activation.

**TABLE 1:** Transcriptional regulation of Arabidopsis genes by volatile organic compounds (VOCs) and their poplar orthologs responsive to ectomycorrhiza symbiosis.

Gene name	Gene function	AGI	Regulation	Host	Treatment	Experimental set-up	Sample tissue	References
*GSTU1*	Glutathione S-transferase TAU 1, responsive to ME-JA	AT2G29490	Up	*P. x canescens*	*L. bicolor*	Pot, in root contact	Leaves	Kaling et al., 2018
			Up	*A. thaliana*	Monoterpene isolated from plants	Pot, exposed to Ocimene	Rosette leaves, stems, cauline leaves	Godard et al., 2008
*GSTU4*	Glutathione S-transferase tau 4, involved in defense from necrotrphic pathogens	AT2G29460	Up	*P. x canescens*	*P. involutus*	Pot, in root contact	Roots	Luo et al., 2009
			Up	*A. thaliana*	Monoterpene isolated from plants	Pot, exposed to Ocimene	Rosette leaves, stems, cauline leaves	Godard et al., 2008
*JAZ1*	JAZ1, involved in jasmonate signaling, defense, wounding. JAZ1 transcript levels rise in response to a jasmonate stimulus.	AT1G19180	Down	*P. x canescens*	*L. bicolor*	Pot, in root contact	Leaves	Kaling et al., 2018
			Up	*A. thaliana*	Monoterpene isolated from plants	Pot, exposed to Ocimene	Rosette leaves, stems, cauline leaves	Godard et al., 2008
*JAZ7*	Jasmonate-zim-domain protein 7; wounding response	AT2G34600	Up	*A. thaliana*	Monoterpene isolated from plants	Pot, exposed to Ocimene	Rosette leaves, stems, cauline leaves	Godard et al., 2008
			Down	*A. thaliana*	1-decene isolated from Trichoderma	Plants in petri dish, 1-decene added	shoots	Lee et al., 2019
*JAZ8*	Jasmonate-zim-domain protein 8; wounding response	AT1G30135	Down	*P. x canescens*	*L. bicolor*	Pot, in root contact	Leaves	Kaling et al., 2018
			Down	*A. thaliana*	1-decene isolated from Trichoderma	Plants in petri dish, 1-decene added	Shoots	Lee et al., 2019
*WRKY40*	Probable WRKY transcription factor 40; Pathogen-induced transcription factor, response to chitin, SA, Me-JA	AT1G80840	Up	*P. x canescens*	*P. involutus*	pot, in root contact	Roots	Luo et al., 2009
			Up	*A. thaliana*	Monoterpene isolated from plants	Pot, exposed to Ocimene	Rosette leaves, stems, cauline leaves	Godard et al., 2008
			Down	*A. thaliana*	1-decene isolated from Trichoderma	Plants in petri dish, 1-decene added	Shoots	Lee et al., 2019
			Up	*A. thaliana*	Monoterpene isolated from plants	Pot, exposed to Pinene	Leaves	Riedlmeier et al., 2017
*SPX1*	SPX domain-containing protein 1; response to phosphate starvation, response to *Pseudomonas syringae*	AT5G20150	up or down (depending on poplar homolog)	*P. x canescens*	*P. involutus*	Pot, in root contact	Roots	Luo et al., 2009
				*A. thaliana*	Rhizobacteria	Bi-compartmented petri dishes, no contact	Seedlings	Wenke et al., 2012
*PAP1*	Purple acid phosphatase, response phosphate (Pi) and phosphite (Phi), response to non-host bacteria.	AT1G13750	Down	*P. x canescens*	*P. involutus*	Pot, in root contact	Roots	Luo et al., 2009
			Up	*A. thaliana*	Monoterpene isolated from plants	Pot, exposed to Pinene	Leaves	Riedlmeier et al., 2017
*BBE8*	FAD-binding Berberine family protein, response avirulent *Pseudomonas synrigae*, response to non-host bacteria	AT1G30700	Up	*P. x canescens*	*P. involutus*	Pot, in root contact	Roots	Luo et al., 2009
			Up	*A. thaliana*	Monoterpene isolated from plants	Pot, exposed to Pinene	Leaves	Riedlmeier et al., 2017
*-*	Putative β-1,3-endoglucanase, response to nematode, response to fungus	AT4G16260	Up	*P. x canescens*	*L. bicolor*	Pot, in root contact	Leaves	Kaling et al., 2018
			Up	*A. thaliana*	Monoterpene isolated from plants	Pot, exposed to Pinene	Leaves	Riedlmeier et al., 2017
*PRX47*	Peroxidase superfamily protein, response to oxidative stress	AT4G33420	Down	*P. x canescens*	*L. bicolor*	Pot, in root contact	Leaves	Kaling et al., 2018
			Up	*A. thaliana*	Monoterpene isolated from plants	Pot, exposed to Pinene	Leaves	Riedlmeier et al., 2017
*-*	Tetratricopeptide repeat (TPR)-like superfamily protein	AT4G37380	Down	*P. x canescens*	*P. involutus*	Pot, in root contact	Roots	Luo et al., 2009
			Down	*A. thaliana*	Monoterpene isolated from plants	Pot, exposed to Pinene	Leaves	Riedlmeier et al., 2017
*SIS*	Salt Induced Serine rich, response to salt, response to virulent *Pseudomonas syringae*	AT5G02020	Up	*P. x canescens*	*P. involutus*	Pot, in root contact	Roots	Luo et al., 2009
			Up	*A. thaliana*	Monoterpene isolated from plants	Pot, exposed to Pinene	Leaves	Riedlmeier et al., 2017
*KAT5*	3-keto-acyl-CoA thiolase 2 precursor, involved in flavonoid biosynthesis	AT5G48880	Up	*P. x canescens*	*L. bicolor*	Pot, in root contact	Leaves	Kaling et al., 2018
			Down	*A. thaliana*	Monoterpene isolated from plants	Pot, exposed to Pinene	Leaves	Riedlmeier et al., 2017
*CCT101*	Member of ASML2 family of CCT domain proteins, high expression in eds16 mutants (isochorimate synthase for SA synthesis)	AT5G53420	Down	*P. x canescens*	*L. bicolor*	Pot, in root contact	Leaves	Kaling et al., 2018
			Down	*A. thaliana*	Monoterpene isolated from plants	Pot, exposed to Pinene	Leaves	Riedlmeier et al., 2017
*CYP94B1*	cytochrome P450, family 94, subfamily B, polypeptide 1, JA metabolic process, wounding	AT5G63450	Down	*P. x canescens*	*L. bicolor*	Pot, in root contact	Leaves	Kaling et al., 2018
			Down	*A. thaliana*	Monoterpene isolated from plants	Pot, exposed to Pinene	Leaves	Riedlmeier et al., 2017

*The table summarizes differentially expressed genes overlapping between VOC and EMF response. AGI shows Arabidopsis Gene Identity for the best poplar match. Treatment indicates the EMF used for plant inoculation or the VOC to which plants were exposed to. Experimental set-up indicates non-sterile conditions when plants were grown in pots or sterile growth systems. The gene functions were taken from the TAIR data base (https://www.arabidopsis.org/) and response were also searched via the eFP browser implemented in TAIR.*

### Mycorrhiza Induced Resistance in Systemic Tissues—Signals and Defense Activation

Phytohormones orchestrate the expression of defense-related genes in systemic tissues. In response to biotrophic pathogens, accumulation of SA is accompanied by the induction of *PR* (Pathogenesis-related) gene expression (Dixon et al., 1994; Hammond-Kosack and Jones, 1997; Brodersen et al., 2005; Radojičić et al., 2018). The most prominent representative of the PR proteins is PR1, which is characteristic for the SA defense pathway (Nimchuk et al., 2003; Durrant and Dong, 2004; Glazebrook, 2005). AMF can activate SA defenses in their host plants (Barea and Jeffries, 1995; García-Garrido and Ocampo, 2002). AMF-colonized crops exhibit enhanced resistance against *Phytophthora infestans* (potato, Gallou et al., 2011), *Magnaporthe oryzae* (rice, Campos-Soriano et al., 2012), and *Alternaria solani* (tomato, Song et al., 2015). The defense induction was attributed to MIR by AMF (Table 2) and has similarities with SAR (see section “A Glimpse on Systemic Resistance in Plants—SAR and ISR”).

**TABLE 2:** Systemic defense activation by mycorrhizal plants.

Gene name	Gene function	Proposed defense pathway	Mycorrhiza Type	Mycorrhiza species	Effects of mycorrhiza	Plant host	Resistance against	Disease/Effect	References
*PMR4*	Callose synthase	JA pathway	AMF	*Rhizoglomus irregularis*	Fungal biomass- *B. cinerea* reduced to 66%	Tomato- *Solanum lycopersicum*	Fungus- *Botrytis cinerea*	Gray mold	Sanmartín et al., 2020
*ATL31*	Carbon/Nitrogen insensitive 1(Arabidopsis Toxicos en Levadura 31)								
*SYP121*	Vesicular trafficking protein								
*VCH3*	Chitinase	Chitinase induced defense pathway	AMF	*Glomus versiforme*	Significant reduction in *M. incognita* infection	Grapevine- *Vitis amurensis*	Nematode- *Meloidogyne incognita*	Root knot	Li et al., 2006
*CHI*	Chitinase 1b	JA and SA pathway	AMF	*Glomus intraradices*	*X. index* count in soil and galls reduced significantly (after 35 days)	Grapevine- *Vitis berlandieri × Vitis riparia*	Nematode- *Xiphinema index*	Root gall	Hao et al., 2012
*PR10*	Pathogenesis-related 10								
*GST*	Glutathione S-transferase								
*STS*	Stilbene synthase 1								
*ESPS*	5-enolpyruvyl shikimate-3-phosphate synthase								
*PR1-a*	Pathogenesis-related 1	SA pathway	AMF	*Glomus mosseae*	74—84% decrease in necroses andintraradical pathogen hyphae of *P. phytophthora*	Tomato- *Solanum lycopersicum*	Pathogen- *Phytophthora parasitica*	Fruit rot	Cordier et al., 1998
*OsNPR1*	Non-expressor of PR1	JA and SA pathway	AMF	*Glomus intraradices*	Significant reduction in spore count of *M. oryzae*	Rice- *Oryza sativa L.*	Fungus- *Magnaporthe oryzae*	Rice blast	Campos-Soriano et al., 2012
*OsAP2*	APETALA2								
*OsEREBP*	Ethylene-responsive element-binding protein								
*OsJAmyb*	JA-regulated myb transcription factor								
*PR*	Pathogenesis-related								
*PR2a*	Pathogenesis-related 2a	DIMBOA- phytoalexin based defense and JA pathway	AMF	*Glomus mosseae*	Disease index of *R. solani* reduced by 50%	Corn- *Zea mays*	Fungus- *Rhizoctonia solani*	Sheath blight	Song et al., 2011
*PAL*	Phenylalanine ammonia-lyase								
*AOS*	Allene oxide synthase								
*BX9*	DIMBOA (2,4-dihydroxy-7-methoxy-2 H-1,4-benzoxazin-3(4 H)-one) biosynthesis pathway gene								
*PR1, PR2*	Pathogenesis-related 1, pathogenesis-related 2	SA pathway	AMF	*Glomus* sp.	Leaf infection index decreased significantly.	Potato- *Solanum tuberosum*	Pathogen- *Phytophthora infestans*	Late blight	Gallou et al., 2011
*POX381*	Peroxidase	SA pathway	AMF	*Funneliformis mosseae*	*B. graminis* infection on leaves reduced to 78%.	Wheat- *Triticum* sp.	Fungus- *Blumeria graminis f.* sp. *Tritici*	Powdery mildew	Mustafa et al., 2017
*PAL*	Phenylalanine ammonia lyase								
*CHI1*	Chitinase 1								
*NPR1*	Non-expressor of pathogenesis-related proteins 1								
*PAL*	Phenylalanine ammonia lyase	JA pathway	AMF	*Glomus Macrocarpum; Glomus Fasciculatum*	*F. oxysporum* disease severity reduced to ∼ 75%	Tomato- *Solanum lycopersicum*	Fungus- *Fusarium Oxysporum f.* sp. *Lycopersici*	Fusarium wilt	Kapoor, 2008
*LOX*	Lipoxygenase	JA pathway	AMF	*Glomus fasciculatum*	Significant decrease in the severity of fusarium wilt disease.	Tomato- *Solanum lycopersicum*	Fungus- *Fusarium Oxysporum f.* sp. *Lycopersici*	Fusarium wilt	Nair et al., 2015
*LOX*	Lipoxygenase	JA pathway	AMF	*Glomus fasciculatum*	Decrease in disease severity of *A. alternata*	Tomato- *Solanum lycopersicum*	Pathogen-*Alternaria alternata*	Fruit rot	Nair et al., 2015
*OPR3*	12-oxophytodienoate reductase 3								
*COI1*	Coronatine-insensitive1								
*PR1, PR2, PR3*	Pathogenesis related1, Pathogenesis related 2, Pathogenesis related 3	JA and SA pathway	AMF	*Funneliformis mosseae*	Disease index of *A. solani* reduced by 54.3%	Tomato- *Solanum lycopersicum*	Pathogen- *Alternaria solani*	Early blight	Song et al., 2015
*LOX*	Lipoxygenase								
*AOC*	Allene oxide cyclase								
*PAL*	Phenylalanine ammonia-lyase								
*LOXD*	Lipoxygenase D	JA pathway	AMF	*Glomus mosseae*	62.3% less weight gain of *H. arimegera* larvae	Solanum lycopersicum Mill.	Insect- *Helicoverpa arimigera*	Herbivory	Song et al., 2013
*AOC*	Allene oxide cyclase								
*PI-I and PI-II*	Serine protease inhibitors I and II								
*AOS1*	Allene oxide synthase 1	JA pathway, phenylpropanoid pathway and protease inhibitor activity	AMF	*Rhizophagus irregularis*	Larvae weigh ∼40 mg which is significantly lower than the control (after 8 days).	Potato- *Solanum tuberosum*	Insect- *Trichoplusia ni*	Herbivory	Schoenherr et al., 2019
*OPR3*	12-oxo-phytodienoate reductase 3								
*PI-I*	Protease inhibitor type								
*PAL*	Phenylalanine ammonia lyase								
JAZ(JAR1, JAR8)	Jasmonate zim domain 1	JA pathway	EMF	*Laccaria bicolor*	Significant less oviposition by beetles on mycorrhizal host plant.	Poplar- *Populus × canescens*	Insect- *Chrysomela populi*	Herbivory	Kaling et al., 2018
MYB (MYB4, MYB5, MYB14 and MYB108)	Transcription factors of JA								
*NAS3*	Nicotianamine synthase								
*KPI*	Kunitz protease inhibitors								
*CHI*	Chitinases								
*CERK1*	Chitin receptor	Both JA and SA pathway	EMF	*Laccaria bicolor*	27% reduction in larval weight on *L. bicolor* colonized host plant.	Arabidopsis	Insect- *Trichoplusia ni*	Herbivory	Vishwanathan et al., 2020
*PR1, PR2 and PR5*	Pathogenesis related 1, Pathogenesis related 2, Pathogenesis related 5	SA pathway	Endophyte	*Piriformospora indica*	33—59% reduction in colony numbers of *B. graminis*	Barley- *Hordeum vulgare*	Fungus- *Blumeria graminis f.* sp. *Hordei*	Powdery mildew	Molitor et al., 2011
*Hsp70, Hsp17.9*	Heat shock proteins70; Heat shock proteins17.9								
*BCI-7*	Barley chemically induced 7								
*PR1, PR5*	Pathogenesis-related 1, Pathogenesis-related 5	JA and SA pathway	Endophyte	*Piriformospora indica*	∼ 50% reduction in the number of conidia of *G. orontii* formed per mycelium.	Arabidopsis	Fungus- *Golovinomyces orontii*	Powdery mildew	Stein et al., 2008
*ERF1*	Ethylene response factor 1								
*PDF1.2*	Plant defensin 1.2								
*VSP*	Vegetative storage protein								

*Symbiotic relationships between mycorrhiza and plants were reviewed with regard to the defense induction by mycorrhiza. Genes were grouped upon their function as well as their effect on the disease of pathogens on their hosts.*

Likewise, the EMF-induced systemic resistance also involves components of SAR signaling. The fitness of caterpillars feeding on SAR signaling mutants of Arabidopsis (*npr1, npr3/4*) was reduced, similar to the effects imposed by *L. bicolor* inoculation (Vishwanathan et al., 2020). In poplar leaves, transcriptional regulation of *NPR1* was detected in EMF-colonized compared to non-colonized plants (Kaling et al., 2018). Pfabel et al. (2012) observed enhanced levels of SA in poplars colonized by the EMF *Hebeloma mesophaeum* as well as in poplars challenged with rust fungi *Melampsora larici-populina.* Therefore, it is likely that similarly to AMF, EMF systemically activate components of the SAR pathway (Figure 1).

In AMF, the induction of down-stream defenses against pathogens is often less pronounced than by SAR and therefore, the alerted stage induced by mycorrhizal colonization has been considered as “priming” (Cameron et al., 2013). As defined by Pozo and Azcón-Aguilar (2007), the phenomenon of priming is the pre-conditioning of the plant host for a more efficient activation of plant defenses upon pathogen attack (Jung et al., 2012). “Priming” by AMF involves, for instance, transcriptional regulation of *PR1* and *NPR1*, hallmarks of the SA pathway (Cameron et al., 2013). However, AMF also prime the JA pathway in the host plant as an “alert” signal against necrotrophic pathogens and leaf-chewing insects (Glazebrook, 2005; Pozo and Azcón-Aguilar, 2007; Jung et al., 2012). These responses include transcriptional regulation of *MYBs* (many of these transcription factors are induced by JA), *LOX* (*LIPOXYGENASE*), *OPR* (*12−OXOPHYTODIENOATE REDUCTASE*), *COI* (*CORONATINE−INSENSITIVE*), *AOC* (*ALLENE OXIDE CYCLASE*), and *AOS* (*ALLENE OXIDE SYNTHASE*) etc. (Table 2). While most studies tested alleviation of damage by necrotrophic pathogens (Table 2), increased resistance against herbivores such as cabbage looper (*Trichoplusia ni*) and cotton bollworm (*Helicoverpa armigera*) was also reported for AMF crops (Song et al., 2013; Schoenherr et al., 2019).

Ecological studies often show beneficial effects of EMF-colonization on the resistance of tree species from different habitats and different phylogenetic origin, e.g., *Larix sibirica, Betula pubescens*, and *Eucalyptus urophylla* against herbivores (*Otiorhyncus* spp., *Anomala cupripes*, and *Strepsicrates* spp. (Halldórsson et al., 2000; Gange et al., 2005; Shen et al., 2015). For example, on the leaves of birch, the birch aphid *Calaphis flava* produces significantly less nymphs when the trees are colonized with EMF (*Paxillus involutus* or *Leccinum versipelle*) compared with non-mycorrhizal trees (Nerg et al., 2008). However, beneficial effects of EMF on the host are not always observed. Larval growth of the autumnal moth *Epirrita autumnata* was not attenuated on EMF-colonized birch trees (Nerg et al., 2008) and EMF colonization of pine roots had no effect on the oviposition of generalist herbivore *Lygus rugulipennis* (Manninen et al., 1998). These studies suggest that the resistance induced by EMF is context-dependent. This idea is also supported by recent transcriptome analyses showing that host defense gene expression of leaves can be diminished when the tree roots are colonized by EMF and depends on the specific host—EMF combination (Maboreke et al., 2016; Bacht et al., 2019; Bouffaud et al., 2020).

**FIGURE 2:**
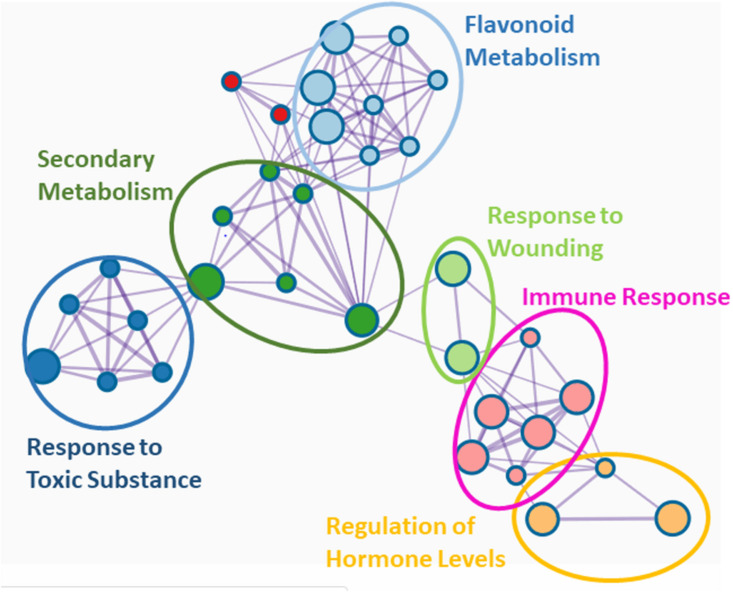
Network of GO terms in systemic leaves after ectomycorrhizal colonization of poplar. Data of differentially expressed genes (DEGs) between mycorrhizal and non-mycorrhizal trees were taken from the Supplemental Table S1 in Kaling et al. (2018). It should be noted that many DEGS were down regulated. The best matches of Arabidopsis orthologs of the poplar genes were uploaded and analyzed in Metascape (Zhou et al., 2019). Significant GO terms (*P*_adjusted_ < 0.05) are shown.

Genetic studies exploring the systemic consequences of EMF-plant interaction are scarce. Arabidopsis knock-out mutants of *coi1-16*, which cannot activate the JA pathway, are more susceptible to cabbage looper feeding than the wildtype, indicating that the protective effect of *L. bicolor* is lost when the JA signaling is compromised (Vishwanathan et al., 2020). In poplar, *L. bicolor* induced a transcriptional network characterized by six major gene ontology (GO) terms: “regulation of phytohormones,” “immune response,” “response to wounding,” “flavonoid metabolism,” “secondary metabolism,” and “response to toxic substance” (Figure 2). “Regulation of phytohormones” and “immune response” comprise mainly transcription factors such as *JAZ1* (orthologs of *JAR1* and *JAR8*) and *MYBs* (orthologs of *MYB4, MYB5, MYB14*, and *MYB108*) which are key the regulators of the JA responses (Goossens et al., 2016). Altogether, these studies imply that regulation of MIR by EMF involves both JA and SA signaling pathways (Figure 1).

Induction of JA and SA-related gene expression also occurs in beneficial fungi, which do not form mycorrhizal structures such as *Serpendita indica* (formerly known as *Piriformospora indica*, Sebacinales, Basidiomycota), and *Trichoderma* sp. (Basidiomycota) (Table 2). *Serpendita indica* activates *PR1* as well as PDF1.2 (defensin) expression in its host (Stein et al., 2008; Molitor et al., 2011). *Trichoderma harzianum* induces JA- and SA-dependent defenses against *Botrytis cineria* by stimulating defense proteins such as PROTEINASE INHIBITOR II and MULTICYSTATIN (Martinez-Medina et al., 2013). *Trichoderma* sp., which is available as commercial inoculum, has often been reported to be a potent biocontrol agent against pathogens (Sharon et al., 2011; Kumar and Ashraf, 2017). For example, in cucumber *Trichoderma harzianum* caused an increased expression of defense genes [*PR*4, *LOX* (lipoxygenase), *GOL* (galactinol synthase)] against the damping-off disease caused by the pathogen *Phytophthora melonis* (Sabbagh et al., 2017). Similar responses were also observed for the AMF *Glomus mosseae*, suggesting that both are effective in diminishing diseases (Sabbagh et al., 2017). Under field conditions, it is also possible that the induction SA and JA-dependent defenses is the consequence of an interaction of AMF (inducing SA defenses) and beneficial rhizobacteria (inducing JA defense) (Cameron et al., 2013). Similar interactions are feasible for EMF and mycorrhizal helper bacteria, which might be able to boost plant tolerance by growth stimulation (Labbé et al., 2014; Zhao et al., 2014).

### Mycorrhiza Induced Resistance in Systemic Tissues—Preparing the Weapons

In practical terms, the production of defense enzymes and defense metabolites including VOCs are important for enhanced resistance. Enzymes such as peroxidases (PRX), polyphenol oxidases, and laccases and their substrates (phenolic compounds) are important to strengthen the cell wall, thereby, erecting barriers against the spreading of pathogens (Carroll and Hoffman, 1980; Darvill and Albersheim, 1984; Baldwin, 1988). Other enzymes (defensins, chitinases, etc.) have antibiotic activities by attenuating pathogens’ growth (Freeman and Beattie, 2008; War et al., 2012). In poplar colonized by *L. bicolor* the transcript levels of putative chitinases (Kaling et al., 2018) and in *Eucalyptus* colonized by *Pisolithus tinctorius* the activity of chitinases were increased in systemic leaves (Albrecht et al., 1994c; Figure 1). Chitinases hydrolyze glycosidic bonds of chitin, a constituent of the insect exoskeletons and thereby, affect the fitness of herbivores or pathogenic fungi.

An important class of proteins acting as a biocidal compounds against insect-herbivores are the protease inhibitors (PIs) (Conconi et al., 1996; Lawrence and Koundal, 2002; Kim et al., 2009; Dunse et al., 2010). Proteases are vital gut enzymes of insects. PIs disturb the activity of proteases, thus, reducing the overall fitness of herbivorous insects (Zhu-Salzman and Zeng, 2015). PIs have also antimicrobial activities inhibiting the physiological development of pathogens (Jashni et al., 2015). EMF colonization of poplar results in upregulated transcription of Kunitz Trypsin Inhibitors (KTI, a class of PIs) and is accompanied by negative consequences for oviposition (Kaling et al., 2018). AMF colonization of crop plants (potato, tomato) affects *PI* expression, leads to reduced diet quality for larvae of *Trichoplusia ni* and *Helicoverpa armigera*, and reduced growth of the caterpillars (Song et al., 2013; Schoenherr et al., 2019). Therefore, we speculate that PIs are part of the systemically induced defense, irrespective of the mycorrhizal type.

Enzymes commonly induced for biotic defense and involved in MIR are the LOXs (lipoxygenases) (Feussner and Wasternack, 2002; Kawano, 2003; La Camera et al., 2004; Shah, 2005; Baysal and Demirdöven, 2007). LOXs catalyze the hydroperoxidation of polyunsaturated fatty acids (Rosahl, 1996). The resulting hydroperoxides are used as substrates by AOS activating JA-based defenses or by hydroperoxide lyase stimulating “volatile phytoalexins” production (Bate and Rothstein, 1998; Wasternack, 2007; Bruinsma et al., 2009; Lyons et al., 2013; Zhou et al., 2014). In AMF colonized tomato plants upregulation of LOX is associated with defense responses against fungal pathogens (*Alternaria solani*, *Alternaria alternata*, *Fusarium oxysporum*) and cotton bollworm (Song et al., 2013, 2015; Nair et al., 2015).

EMF colonization of roots does not only trigger defense proteins but also results in changes of the leaf metabolome (Pfabel et al., 2012; Cameron et al., 2013; Adolfsson et al., 2017; Hill et al., 2018; Kaling et al., 2018). The compounds mainly involved in enhancing plant tolerance or resistance can be chemically categorized as terpenes, phenolic compounds, nitrogenous, and sulfurous compounds (Mazid et al., 2011; Pedone-Bonfim et al., 2015; Wink, 2018). The terpenes and terpenoids comprise a large class of plant metabolites. Many of these compounds are VOCs, which increase drastically in response to herbivory (“Herbivore-Induced Plant Volatiles”, Pieterse et al., 2014). VOCs act as repellents for herbivores or as attractants to other arthropods that prey upon or parasitize herbivores (Loreto and Schnitzler, 2010). These ecologically important VOCs are produced by the plant down-stream the JA signaling pathway (Ament et al., 2004; van Schie et al., 2007). Since a role of VOCs for plant-insect interactions has often been reviewed (Holopainen and Gershenzon, 2010; Bouwmeester et al., 2019), we illustrate this area just by few selected examples: β-ocimene (monoterpene) and β-caryophyllene (sesquiterpene) emissions are enhanced by AMF-colonized bean plants and recruit natural predators of spider mites (Schausberger et al., 2012). In tomato, AMF colonization enhanced terpene levels and defenses against larvae of the beet armyworm (Shrivastava et al., 2015).

Phenolic compounds are part of the plant defense arsenal and often higher in EMF than in non-mycorrhizal plants (Gange and West, 1994; Baum et al., 2009; Fontana et al., 2009; Schweiger et al., 2014). While phenol-based compounds enhance antibiosis, e.g., against rust (Pfabel et al., 2012), they are not effective against adapted herbivores such as lepidopteran species feeding on Salicaceae (Lindroth and St. Clair, 2013; Boeckler et al., 2014). For example, poplar leaf beetle (*Chrysomela populii*) prefers phenolic-rich leaves (Behnke et al., 2010). Therefore, transcriptional down-regulation of enzymes required for production of secondary compounds (e.g., tannins, flavonoids, phenolic glycosides, proanthocyanidin dimers, and trimers) in EMF-colonized poplar and upregulation of aldoxime production suggests that MIR triggers a metabolic shift from carbon-based to N-based defense (Kaling et al., 2018). Aldoximes and other nitrile-derived compounds are very effective herbivore repellents (Irmisch et al., 2013, 2014; McCormick et al., 2014). The changes induced in systemic tissues by EMF are often subtle or unfold only after biotic attack. Therefore, it will be important to enhance research with a wider range of model systems such as poplar, oak, and conifers, etc., that are amenable to functional studies by transgenic approaches and can be handled under controlled conditions.

## Conclusion

The field of EMF-induced systemic resistance is still scattered but putting the puzzle pieces together, a picture is starting to emerge. EMF in contact with local tissue (roots) activate systemic induced resistance via chitin receptors in Arabidopsis. Since Arabidopsis is a non-host for any mycorrhizal interaction, it will be important to show whether chitin also plays a fundamental role in triggering MIR in EMF-host interactions. EMF-host interactions often positively influence resistance against biotrophic pathogens and herbivory in leaves. The nature of long-distance signaling from roots to leaves remains elusive. Besides vascular routes, aerial transmission via VOCs cannot be excluded (Ditengou et al., 2015). In addition to other effects, systemic leaves show suppressed expression of JAZ transcription factors, whereby transcription of defense proteins and enzymes for the production of defense metabolites is set on. Collectively, studies on AMF or EMF inoculated plants point to activation of JA-related pathways. Still, the recruitment of SA-related defense cannot be dismissed because an involvement of NPR1 and NPR3/4 (positive and negative regulators of SA) has been shown for EMF-induced systemic resistance. The defense responses are versatile. Most of our current knowledge on EMF-activated defenses stems from poplar. Since poplars can be colonized by both EMF and AMF (Khasa et al., 2002; Liu et al., 2015), an increased understanding of MIR requires comparative studies of AMF- and EMF-induced systemic resistance in this host species as well as additional investigations with tree species that can only be colonized by EMF. Since different tree species exhibit a vast range of secondary compounds, there is much work ahead to better understand pathways, which stimulate tree-specific defenses. This is an important task for the future. Since climate change is affecting plant-pest interactions for the worse (Linnakoski et al., 2019), more insights into resistance mechanisms are urgently needed to guide tree selection and breeding for stable future forests.

## Author Contributions

AP conceived the study, supervised writing, and revised the manuscript. SD led the writing. ID, KK, and MJ contributed sections to the manuscript. All authors read and approved the final submission.

## Conflict of Interest

The authors declare that the research was conducted in the absence of any commercial or financial relationships that could be construed as a potential conflict of interest.
